# 1 The Battle of the Titans: Comparing Resuscitation Between 5 Centers Using the Burn Navigator

**DOI:** 10.1093/jbcr/irac012.005

**Published:** 2022-03-23

**Authors:** Julie A Rizzo, Elsa Coates, Jose Salinas, Maria Serio-Melvin, Tam N Pham, Kevin N Foster, Kareem R Abdelfattah, Nehemiah T Liu

**Affiliations:** US Army Institute of Surgical Research, Fort Sam Houston, Texas; US Army Institute of Surgical Research, JBSA Fort Sam Houston, Texas; US Army Institute of Surgical Research, San Antonio, Texas; US Army Institute of Surgical Research, Cibolo, Texas; University of Washington, Seattle, Washington; The Arizona Burn Center Valleywise Health, Phoenix, Arizona; UT-Southwestern Medical Center, Dallas, Texas; U.S. Army Institute of Surgical Research, San Antonio, Texas

## Abstract

**Introduction:**

The goal of burn resuscitation is to provide the least amount of fluid necessary to maintain end-organ perfusion and prevent burn shock. The objective of this analysis was to examine how the Burn Navigator (BN), a clinical decision support tool in burn resuscitation, was utilized across 5 major burn centers in the United States.

**Methods:**

A non-interventional, observational trial of 300 adult patients with embedded prospective and retrospective components was undertaken to examine the effectiveness of the BN in burn resuscitation. 5 ABA-verified burn centers enrolled patients. Data examining patient demographics, burn characteristics, fluid volumes, and resuscitation-related complications were examined. Statistical analysis compared the 5 sites in terms of these variables.

**Results:**

A total of 285 patients were eligible for analysis. There was no difference among the centers in terms of average age (45.5 + 16.8 years), BMI (29.2 + 6.9), ISS (21.2 + 12.8), or median TBSA (34 [25.8, 47]). Primary crystalloid infusion volumes at 24 hours differed significantly when measured in ml/kg/TBSA (median 3.7 [2.9, 8.8], range 1.3 to 12.3). Similarly, total fluids, which includes colloid adjuncts, drip medications and enteral fluids, differed between groups when measured in both ml/kg (median 149.8 [106.5, 224.1], range 38.4 to 536.2) and ml/kg/TBSA (4.2 [3.3, 5.5], 1.7 to 15.3) at 24 hours. Post-hoc adjustment for pairwise comparisons resulted in a loss of significance between most of the sites. There was a total of 156 resuscitation-related complications reported across the 5 sites with an average incidence of 44.4 % incidence.

**Conclusions:**

The Burn Navigator appeared to standardize fluid resuscitations across 5 major US burn centers. With primary fluid volumes near the Parkland formula, the device can be utilized effectively in burn centers, and further study should exam the utility of this device in facilities that do not commonly treat burn injuries, as well as the battlefield.

